# Renoprotective Potential of Beetroot Spent Extract Under Hyperglycemic Conditions

**DOI:** 10.3390/foods15040769

**Published:** 2026-02-20

**Authors:** Wachiraporn Tipsuwan, Onsaya Kerdto, Phronpawee Srichomphoo, Wittaya Chaiwangyen, Pongsak Angkasith, Yanping Zhong, Somdet Srichairatanakool

**Affiliations:** 1 Division of Biochemistry, School of Medical Sciences, University of Phayao, Phayao 56000, Thailand; wachiraporn.ti@up.ac.th (W.T.); wittaya.ch@up.ac.th (W.C.); 2 Department of Biochemistry, Faculty of Medicine, Chiang Mai University, Chiang Mai 50200, Thailand; onsaya35@gmail.com (O.K.); phronpawee0402@gmail.com (P.S.); yanping_z@cmu.ac.th (Y.Z.); 3 Research Excellence, School of Medicine, University of Phayao, Phayao 56000, Thailand; 4 Royal Project Foundation, Chiang Mai 50200, Thailand; pongsak.a@cmu.ac.th; 5 School of Medical Technology and Artificial Intelligence, Youjiang Medical University for Nationalities, Baise 533000, China

**Keywords:** beetroot, *Beta vulgaris*, oxidative stress, reno-protection, hyperglycemia, antioxidant

## Abstract

Diabetic nephropathy is a major complication of diabetes mellitus, primarily driven by hyperglycemia-induced oxidative stress and renal tubular cell injury. Beetroot (*Beta vulgaris* L.) is rich in antioxidant phytochemicals, and its industrial processing generates large amounts of spent material that may retain significant bioactive compounds. This study evaluated the phytochemical profile, antioxidant capacity, and renoprotective potential of beetroot spent extracts under hyperglycemic conditions. Beetroot spent material was extracted using hot water and 70% ethanol. Total phenolic, flavonoid, and betalain contents were quantified, and antioxidant activity was assessed using the 2,2′-azino-bis(3-ethylbenzothiazoline-6-sulfonic acid) (ABTS) radical scavenging assay. Phytochemical characterization was performed by ultra-high-pressure liquid chromatography–electrospray ionization–quadrupole time-of-flight mass spectrometry (UHPLC-ESI-QTOF-MS). Cytotoxicity was evaluated in peripheral blood mononuclear cells, SH-SY5Y, HEK-293, and MDA-MB-231 cells using the 3-(4,5-dimethylthiazol-2-yl)-2,5-diphenyltetrazolium bromide (MTT) assay. Renoprotective effects were investigated in HEK-293 renal tubular cells cultured under normal (5.5 mM) and high-glucose (200 mM) conditions. UHPLC-ESI-QTOF-MS was used to identify over 80 phenolic and flavonoid compounds including quercetin, epicatechin, and epigallocatechin gallate. The hot water extract exhibited superior antioxidant activity, achieving approximately 90% ABTS radical inhibition. Beetroot spent extract showed no cytotoxicity at concentrations below 1 mg/mL and significantly restored HEK-293 cell viability (>90%) under high-glucose conditions at concentrations ≥31.25 µg/mL. In conclusion, beetroot spent water extract possesses strong antioxidant and renoprotective activities against hyperglycemia-induced renal cell damage, supporting its valorization as a sustainable functional food ingredient for diabetes-related health applications.

## 1. Introduction

Diabetic nephropathy is one of the most prevalent and severe microvascular complications of diabetes mellitus and remains a leading cause of chronic kidney disease and end-stage renal failure worldwide [1,2]. Persistent hyperglycemia promotes excessive production of reactive oxygen species (ROS), leading to oxidative stress, mitochondrial dysfunction, inflammation, and progressive damage to renal tubular cells [3]. Although hemodialysis is an effective treatment for advanced renal failure, it can significantly reduce one’s quality of life and place a substantial physical, psychological, and economic burden on patients and their families [4]. Therefore, identifying dietary or natural interventions that can mitigate oxidative stress and protect renal cells under hyperglycemic conditions is of considerable clinical importance.

Beetroot (*Beta vulgaris* L.), a member of the Amaranthaceae family, is widely consumed as a vegetable and is recognized for its rich content of bioactive phytochemicals. In addition to phenolic acids and flavonoids, beetroot is a major source of betalains, including betacyanins and betaxanthins, which are responsible for its characteristic red and yellow pigmentation [5]. These compounds exhibit strong antioxidant, anti-inflammatory, and cytoprotective properties and have been associated with multiple health benefits such as improved vascular function, reduced oxidative stress, and enhanced metabolic regulation [6,7,8]. Emerging evidence also suggests that beetroot supplementation may improve insulin sensitivity and reduce the risk of diabetes-related complications [9,10]. During beetroot juice processing, large quantities of solid residue or “spent beetroot” are generated and typically discarded as agricultural waste. However, previous studies have shown that many bioactive compounds remain entrapped within the cellular matrix and fibrous components of various plant materials, resulting in a high concentration of phenolics and flavonoids in the spent fraction [11,12]. Valorization of beetroot spent material therefore represents a sustainable approach that aligns with circular bioeconomy principles while providing a potentially rich source of natural antioxidants.

Although beetroot bioactive compounds and juice by-products have been previously investigated, limited information is available regarding the biological activity of beetroot juice processing residues in the context of renal cellular stress. Moreover, the phytochemical complexity and biological effects of beetroot spent extracts prepared using different extraction solvents remain insufficiently characterized. Accordingly, this study focuses on the valorization of beetroot spent material by comprehensively investigating its phytochemical profile, antioxidant activity, cytotoxicity in multiple human cell lines, and reno-protective potential in human cell cultures under normal and high-glucose conditions in vitro.

## 2. Materials and Methods

### 2.1. Chemicals and Reagents

Aluminum chloride (Product number 213330, ≥99.5%), ABTS (Product number A1888, ≥98%), fetal bovine serum (FBS) (Product number F7524), dimethyl sulfoxide (DMSO) (Product number 472301, ≥99.9%), 2 N Folin–Ciocalteu’s phenol reagent (Product number F9252, density 1.240 g/mL), formic acid (Product number 33015, ≥98%), sodium carbonate (Product number 223530, ≥99.5%), 3-(4,5-dimethylthiazol-2-yl)-2,5-diphenyltetrazolium bromide (MTT) (Product number M2128, 98%), penicillin (100 U/mL) and streptomycin (100 μg/mL) (Product number P4458), and Ficoll-Paque (Catalogue number GE17-5442-02) were purchased from Sigma-Aldrich Chemicals Company Limited, Saint Louis, MO, USA. Betalain (Product number B2629), gallic acid (GA) (Product number G7384), quercetin (Q) (Product number Q4951, ≥95%), and 6-hydroxy-2,5,7,8-tetramethylchroman-2-carboxylic acid (Trolox) (Product number M2128, 98%) were obtained from the Sigma-Aldrich Chemicals Company. Organic solvents including acetonitrile and ethanol are the highest pure grade.

### 2.2. Plant Material, Beetroot Extraction and Extraction Yield

Beetroot is an agricultural vegetable product collected from the plantation at Ban Mae Hae in the Mae Wang District, located within the Royal Project area of Chiang Mai Province. The beetroot pulp extract was prepared as follows: fresh beetroot (6.27 kg) was rinsed with clean water and juiced using a juice extractor (Tefal model ZN655, Tefal, Riga, Latvia). The waste or spent was then dried in a hot air oven at 50–55 °C for 1–2 days before being ground to a dry weight of 120.93 g. A fraction (20.18 g) was extracted using hot deionized (DI) water (100 g/L) at 80 °C for 10 min. It was then filtered through muslin cloth and filter paper (Whatman No. 1, cellulose type, 110-mm diameter, Cytiva Company, Wilmington, DE, USA). The filtered beetroot water extract was dried in a vacuum freeze-drying machine (Model 4EC, Grisrithong Company Limited, Ratchaburi, Thailand) under the following conditions: −40 °C, 10–12 Pa, overnight. Another fraction (20.0 g) was extracted with 70% (*v*/*v*) ethanol, and the ethanol extract was evaporated using a rotary evaporator (Heidolph HEI-VAP, Becthai Bangkok Equipment and Chemicals Company Limited, Nakhon Pathom, Thailand) at 50–55 °C. Both spent extracts were then stored in aluminum foil bags for future investigation and analysis. Beetroot spent extractions using hot water and 70% ethanol were carried out independently in triplicate, and the resulting extracts were combined for subsequent analyses. Accordingly, fresh beetroot (6.27 kg) yielded 3.13 kg of juice and 3.14 kg of spent material after juicing. Following drying, 120.93 g of dry spent material was obtained. Hot water extraction yielded 3.07 g (15.20%), while 70% ethanolic extraction yielded 2.98 g (14.71%) of dry extract.

### 2.3. Identification of Phytochemical Constituents by UHPLC-ESI-QTOF-MS

UHPLC-ESI-QTOF-MS analysis enabled the putative identification of phytochemicals in the beetroot spent extract (10 mg/mL) under the following conditions: mobile phase A (0.1%, *v*/*v*, formic acid) and mobile phase B (acetonitrile), with a flow rate of 0.35 mL/minute [13]. The separation column was an InfinityLab Poroshell 120 EC-C18 type, measuring 2.1 mm × 100 mm, with a particle size of 2.7 µm, manufactured by Agilent Technologies in Santa Clara, CA, USA. It was maintained at 25 °C. The Agilent Q-TOF mass spectrometer was equipped with a nitrogen gas nebulization system set to 45 pounds · inch^2^. The flow rate was maintained at 5 L/minute at 300 °C; the gas layer was set to 11 L/minute at 250 °C; the capillary and nozzle voltages were 3.5 kV and 500 V, respectively; and the tracer was configured for the 200–3200 *m*/*z* range. The phytochemicals from the available library were analyzed and characterized using ChemStation^®^ Agilent MassHunter Workstation Software Version B.06.01 (Agilent 6545 Q-TOF-LC/MS system). Therefore, compounds with identical accurate mass and molecular formula but distinct retention times were reported as separate putative isomers, reflecting chromatographically resolved features annotated at Metabolic Standard Initiatives (MSI) level 2 (putative annotation); quantitative determination of individual compounds or relative abundances was not conducted.

### 2.4. Determination of Chemical Compositions

#### 2.4.1. Total Phenolic Content (TPC)

Phenolic concentration was evaluated via the Folin–Ciocalteu reaction. A volume of 100 μL of either GA reference solution (12.5–400 μg/mL) or beetroot extract (100 μg/mL) solution was reacted with 200 μL of Folin–Ciocalteu reagent (10%, *v*/*v*). Next, 800 μL of sodium carbonate solution (700 mM) was added to initiate color development. The reaction mixture was kept at ambient temperature in the absence of light for 30 min before measuring optical density (OD) at 765 nm using a double-beam UV–visible spectrophotometer (Shimadzu Corporation, Nakagyo-ku, Kyoto, Japan). Phenolic content was estimated from a GA calibration plot and reported as mg gallic acid equivalents per g of extract (mg GAE/g) [14]. Results are primarily expressed on a dry weight (DW) basis. Values expressed on a wet weight (WW) basis were calculated by back-conversion to the original fresh beetroot material to enable comparison with beetroot juice and existing literature.

#### 2.4.2. Total Flavonoid Content (TFC)

Flavonoid levels were quantified using an aluminum chloride-based colorimetric procedure. In brief, 250 μL of Q standard (12.5–400 μg/mL) or beetroot extract (100 μg/mL) solution was combined with 50 μL of aluminum chloride solution (10%, *v*/*v*) and 50 μL of potassium acetate (1 M), followed by dilution with 2.15 mL DI water. After standing in the dark at room temperature for 30 min, OD was recorded at 415 nm. Quantification was performed using a Q standard curve, and results were expressed as mg quercetin equivalents per g of extract (mg QE/g) [15]. Similarly, results are primarily expressed in DW values and calculated into WW values.

#### 2.4.3. Total Betalain Content (TBC)

The TBC was determined using a spectrophotometric method as previously described [16]. Briefly, the optical density of the beetroot extract solution (1 mg/mL) was measured at 535 nm using a UV–visible spectrophotometer. Betalain concentration was calculated using Equation (1):

TBC (mg/L) = [OD × MW × 10^3^] (1)

[ε × l]

where OD represents the optical density at 535 nm, the molecular weight (MW) of betalain is 550 g/mol, ε indicates the molar extinction coefficient (60,000 L mol^−1^ cm^−1^), and l refers to the light path (1 cm). TBC was expressed as mg betalain per g of extract. This calculation method is widely applied for estimating total betalain content in beetroot and related plant materials.

### 2.5. Antioxidant Activity Analysis

Antioxidant activity was assessed using the ABTS method as follows: 10 μL of DIwater, the Trolox standard (6.25–100 mg/mL), and the sample itself (25–400 mg/mL) were pipetted into each test tube. Then, 990 μL of the working ABTS solution was added, and the test tubes were shaken until they were fully mixed. The test tube was then placed in a dark room at room temperature for 6 min. The OD was measured at a wavelength of 734 nm using distilled water as a blank and adjusted to zero. The antioxidant activity of the test substance was determined using a Trolox standard curve, and the resulting value was expressed as the inhibitory effect on free radical generation [17].

### 2.6. Ethical Approval and Exemption Statement

The study protocol was reviewed by the Human Ethics Committee of the Faculty of Medicine, Chiang Mai University, Chiang Mai, Thailand, and it was determined to be exempt from full ethical review due to the minimal-risk nature of the procedures involved (Study Code: BIO-2567-0111). The study was conducted in accordance with the Declaration of Helsinki and other relevant institutional guidelines. Written informed consent was obtained from the healthy volunteer prior to blood collection.

### 2.7. Peripheral Blood Mononuclear Cell Isolation

Venous blood (15 mL) was taken from a healthy volunteer (male, age 26 years, and weight 52 kg) and collected into lithium heparin-anticoagulant tubes. Whole blood was carefully layered over Ficoll-Paque solution (density 1.077 g/mL) and centrifuged at 3000 rpm, 25 °C for 15 min to separate the plasma and cellular components. The peripheral blood mononuclear cell (PBMC) layer was collected, washed, and cultured in 96-well plates for subsequent experiments [18].

### 2.8. Cell Culture Conditions

The human neuroblastoma cell line SH-SY5Y (ATCC HTB-11, purchased from Thermo-Fisher Scientific Inc., Waltham, MA, USA) was cultured in DMEM containing 1% glutamine, 10% (*v*/*v*) FBS, and penicillin–streptomycin. Cells were incubated at 37 °C in a humidified atmosphere of 5% CO_2_ and used for experiments when confluence reached 70–80% [19].

Human embryonic kidney 293 (HEK-293; ATCC TRL-1573) cells (purchased from Thermo-Fisher Scientific Inc., Waltham, MA, USA) were grown in DMEM supplemented with 10% (*v*/*v*) FBS and penicillin–streptomycin. Cells were incubated at 37 °C in a humidified atmosphere of 5% CO_2_ and used for experiments when confluence reached 70–80% [20].

The human breast cancer cell line MDA-MB-231 (ATCC HTB-26, purchased from Thermo-Fisher Scientific Inc., Waltham, MA, USA), an aggressive, triple-negative breast cancer cell line lacking an estrogen receptor, a progesterone receptor, and human epidermal growth factor receptor 2, was cultured in DMEM supplemented with 10% (*v*/*v*) FBS and penicillin–streptomycin. Cells were maintained at 37 °C with 5% CO_2_ until 70–80% confluence was reached, before they were used in the experiments [21].

The human hepatocellular carcinoma (Huh7) cell line was purchased from Thermo-Fisher Scientific Inc., Waltham, MA, USA. The cells were maintained in standard glucose media for 24 h [19].

### 2.9. Cytotoxicity Assessment of Beetroot Extracts

Before cell culture treatment, beetroot spent extracts were freshly prepared, dissolved in sterile distilled water or appropriate culture medium, and sterilized by filtration through a 0.22-μm pore-size membrane filter to ensure sterility. The PBMC and Huh7 cells were treated with beetroot extracts (31.25–1000 µg/mL), cultured in a CO_2_ incubator at 37 °C for 24 and 48 h, and subjected to the MTT assay as described below. The SH-SY5Y neuronal and MDA-MB-231 breast cancer cell lines were also included to assess the general cytotoxicity and cellular compatibility of beetroot spent extract across non-renal human cell types, rather than to model diabetic nephropathy. Similarly, the SH-SY5Y and MDA-MB-231 cells were treated with beetroot extracts (31.25–1000 μg/mL), cultured in a CO_2_ incubator at 37 °C for 24 and 48 h, and subjected to the MTT assay.

### 2.10. Colorimetric MTT Assay

The MTT assay was used as an indirect measure of cell viability based on mitochondrial metabolic activity. Cytotoxicity was assessed by adding 0.5 mg/mL of MTT solution to the cultured cells, and they were then incubated at 37 °C for 4 h. After aspirating the cell culture media, 200 μL of DMSO was added to dissolve the purple formazan crystals, and the OD of the solution was measured at 570 nm [19]. The percentage of cell viability was then calculated.

### 2.11. Study of Protective Effect of Beetroot Spent Extract Under Hyperglycemic Conditions

The concentration range was chosen based on preliminary cytotoxicity screening, literature reports on plant-derived extracts in cell culture, and to ensure coverage of non-cytotoxic to potentially bioactive concentrations under high-glucose-associated stress conditions. HEK-293 cells were cultured in DMEM containing normal glucose (5.5 mM) and high-glucose concentrations (6.25–400 mM), 10% (*v*/*v*) FBS, and penicillin–streptomycin in a 37 °C incubator with 5% CO_2_ until they reached 70–80% confluence. The cells were treated with beetroot extract (31.25–1000 μg/mL) cultured for 24 and 48 h, and then the cell viability was assessed using the MTT method, as has been described above [22].

### 2.12. Statistical Analysis

Descriptive statistics were employed to analyze population characteristics in randomly selected groups using SPSS Statistics software version 22 (IBM SPSS Statistics, Armonk, NY, USA). Results are presented as percentages, frequencies, distributions, and means ± standard deviations (SD). Statistical comparisons among multiple groups were performed using the one-way analysis of variance (ANOVA), followed by Tukey’s post hoc multiple comparison test. Comparisons between two groups were performed using Student’s *t*-test. A *p*-value of < 0.05 was considered statistically significant.

## 3. Results

### 3.1. Extraction Yield of Beetroot Juice and Spent Extracts

Results in Table 1 indicate that both extraction methods produced similar yields from beetroot spent material.

### 3.2. Phytochemical Profiling of Beetroot Spent Extract by UHPLC-ESI-QTOF-MS

**Figure 1:**
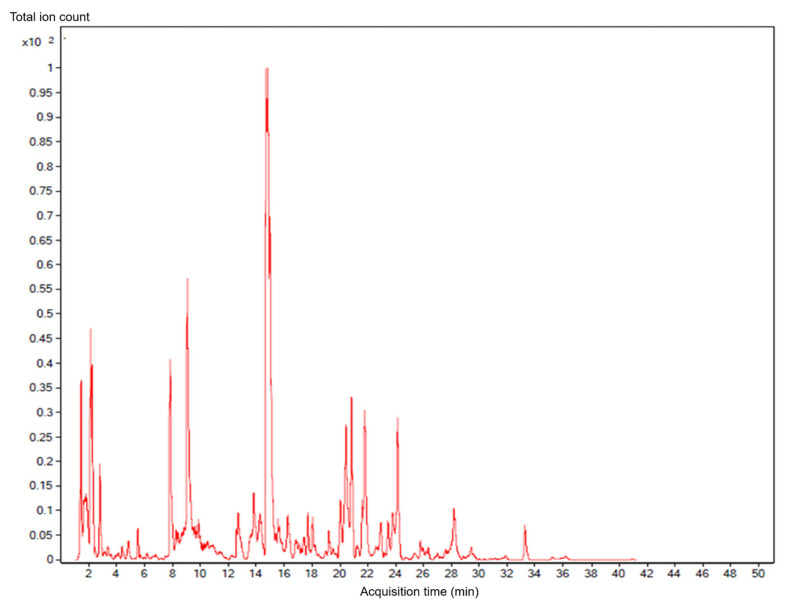
Representative UHPLC-ESI-QTOF-MS chromatogram showing the major phytochemical compounds identified in the hot water extract of beetroot spent material.

The chromatographic analysis revealed a complex phytochemical composition, with numerous phenolic acids, flavonoids, and anthocyanin-related compounds detected (Figure 1). The diversity of the identified compounds indicated that the spent beetroot fraction retained a broad spectrum of bioactive constituents following juice extraction.

The listed compounds represent putatively identified constituents detected by UHPLC-ESI-QTOF-MS; peak area quantification or relative abundance analysis was not performed. Detailed compound identification based on accurate mass measurements and database matching revealed the presence of multiple phenolic and flavonoid compounds in the beetroot spent hot water extract (Table S1). Major identified compounds included flavonoids, such as quercetin, epicatechin, gallocatechin, and epigallocatechin gallate, as well as phenolic acids including caffeic acid, chlorogenic acid, ferulic acid, and p-coumaric acid. Several glycosylated and sulfated derivatives of the phenolics and anthocyanins were also detected, demonstrating the chemical complexity and antioxidant potential of the extract. In addition, several phytochemicals, including chlorogenic acid and epicatechin–(4β ⟶ 8)–gallocatechin, were detected as multiple chromatographic peaks with identical molecular formulae and were therefore reported as putative isomeric forms.

### 3.3. Total Phenolic, Flavonoid, and Betalain Contents of Beetroot Extracts

Quantitative analysis showed that beetroot juice contained a TPC of 12.27 ± 3.03 mg GAE/g DW or 0.24 ± 0.01 GAE/g WW, whereas the hot water and 70% ethanolic extracts of beetroot spent material contained 9.16 ± 1.08 and 5.41 ± 0.72 mg GAE/g DW, or 1.56 ± 0.24 and 0.77 ± 0.07 mg/g WW, respectively (Table 2). The TFC of beetroot juice was 0.52 ± 0.11 mg QE/g DW, or 0.01 ± 0.00 mg QE WW, while hot water and ethanolic spent extracts contained 4.76 ± 0.37 and 0.29 ± 0.05 mg QE/g DW, or 0.86 ± 0.10 and 0.03 ± 0.02 mg QE/g WW, respectively.

In addition, TBC was highest in beetroot juice (4.55 ± 0.01 µg/g DW), followed by the hot water extract of spent beetroot material (0.60 ± 0.00 µg/g DW) and 70% ethanolic extract (0.28 ± 0.00 µg/g DW). Overall, the hot water extract of the beetroot spent material retained substantial levels of phenolic compounds and flavonoids when compared with the ethanolic extract. Notably, DW-based values are emphasized in the interpretation of extraction efficiency, as they directly reflect compound enrichment in the dried extracts.

### 3.4. Antioxidant Activity of Beetroot Extracts Assessed by ABTS Assay

**Figure 2:**
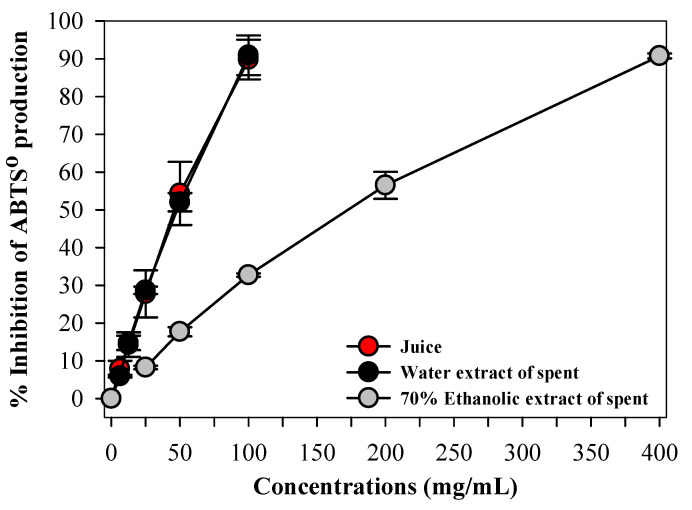
Antioxidant activity of beetroot juice, hot water extract of beetroot spent, and 70% ethanolic extract as determined by the ABTS radical scavenging assay. Results are expressed as percentage inhibition and presented as mean ± SD values (*n* = 3).

The antioxidant activity of beetroot juice and spent extracts was evaluated using the ABTS radical scavenging assay. All extracts exhibited concentration-dependent antioxidant activity (Figure 2). Beetroot juice and the hot water extract of beetroot spent demonstrated markedly stronger ABTS radical inhibition when compared with the 70% ethanolic extract at equivalent concentrations. At higher concentrations, beetroot juice and hot water extract achieved up to approximately 90% inhibition of ABTS radicals, whereas the ethanolic extract exhibited a maximum inhibition of approximately 33%. These results indicate the superior antioxidant capacity of the aqueous beetroot spent extract.

Thus, differences in antioxidant activity among extracts are interpreted in the context of solvent-dependent extraction of hydrophilic antioxidant compounds and the chemical sensitivity of the ABTS assay.

### 3.5. Effect of Beetroot Spent Extract on Cell Viability Under Normal Glucose Conditions

**Figure 3:**
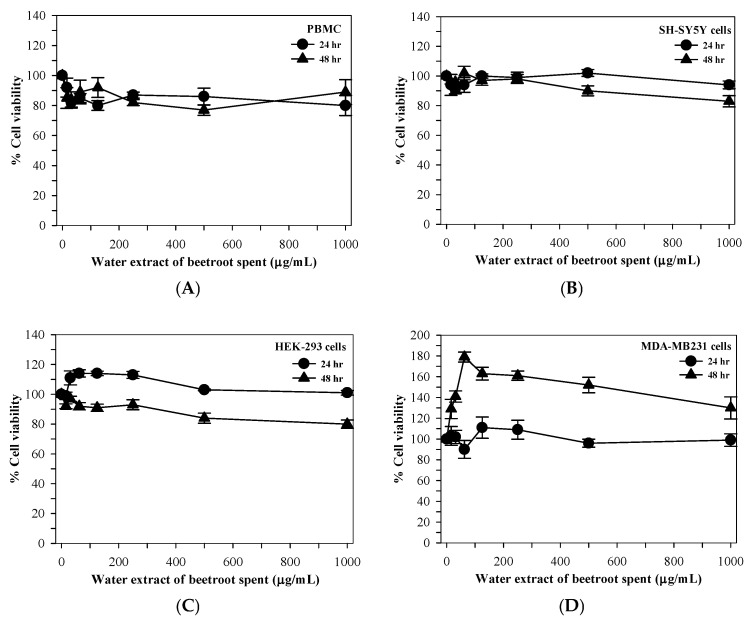
Effects of hot water extract obtained from beetroot spent on cell viability under normal glucose conditions. Cell viability of (**A**) PBMCs, (**B**) SH-SY5Y neuronal cells, (**C**) HEK-293 renal tubular cells, and (**D**) MDA-MB231 breast cancer cells after 24 and 48 h of treatment. Data are presented as mean ± SD values from three independent experiments.

The cytotoxicity of the hot water extract of beetroot spent was evaluated in multiple human cell lines under normal glucose conditions. In PBMCs, extract concentrations below 100 µg/mL did not exhibit cytotoxic effects after 24 or 48 h of treatment, with cell viability remaining above 80% at all tested concentrations (Figure 3A). Similarly, SH-SY5Y neuronal cells exposed to beetroot spent extract for 24 and 48 h maintained high viability (>90%) across all tested concentrations (Figure 3B). HEK-293 renal tubular cells treated with the extract also showed no cytotoxic effects, with cell survival exceeding 85%. Notably, short-term exposure (24 h) resulted in a modest increase in metabolic activity, whereas prolonged exposure (48 h) led to a slight reduction in cell viability (Figure 3C). In MDA-MB231 breast cancer cells, beetroot spent extract did not induce cytotoxicity after 24 h of treatment. While a mild increase in reducing power was observed at 24 h, prolonged exposure (48 h) resulted in a modest decrease in metabolic activity without a significant reduction in overall cell viability (Figure 3D).

### 3.6. Effect of High Glucose Levels on HEK-293 Renal Tubular Cell Viability

**Figure 4:**
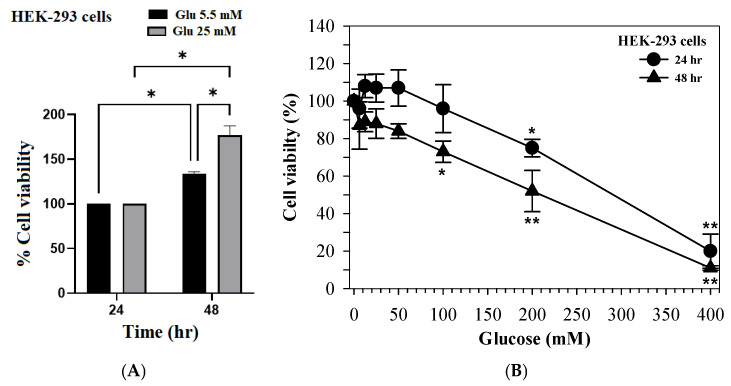
Effect of glucose concentrations on HEK-293 renal tubular cell viability. (**A**) Cell viability under normal (5.5 mM) and moderate (25 mM) glucose concentrations at 24 and 48 h. (**B**) Cell viability under increasing glucose concentrations (50–400 mM). Data are expressed as mean ± SD values (*n* = 3). * *p* < 0.05, ** *p* < 0.01 when compared with the control.

To establish a hyperglycemic injury model, HEK-293 cells were cultured under varying glucose concentrations. Under normal (5.5 mM) and moderately high-glucose (25 mM) conditions, cell viability significantly increased over time, with higher viability observed at 48 h when compared with 24 h (Figure 4A). In contrast, exposure to glucose concentrations exceeding 100 mM resulted in significant cytotoxicity. Accordingly, cell viability decreased in a dose-dependent manner as the glucose concentration increased from 50 to 400 mM (Figure 4B). Notably, HEK-293 cells exposed to high amounts of glucose exhibited lower viability at 24 h when compared with 48 h, indicating acute glucose-induced cytotoxic stress.

### 3.7. Protective Effect of Beetroot Spent Extract Against High-Glucose-Induced Cytotoxicity

**Figure 5:**
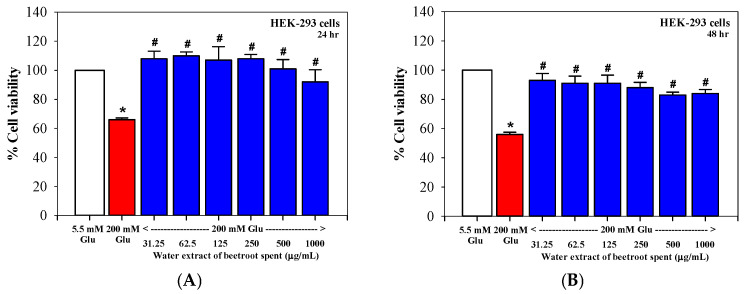
Protective effects of hot water extract obtained from beetroot spent on HEK-293 renal tubular cells cultured under high-glucose conditions (200 mM). Cell viability after (**A**) 24 h and (**B**) 48 h of treatment. Data are presented as mean ± SD values (*n* = 3). * *p* < 0.05 when compared with normal glucose control (5.5 mM); # *p* < 0.05 when compared with high glucose without extract.

Exposure of HEK-293 cells to high glucose concentrations (200 mM) significantly reduced cell viability to below 60% when compared with cells cultured under normal glucose conditions (5.5 mM). Treatment with beetroot spent hot water extract at concentrations ≥31.25 µg/mL significantly improved cell survival under high-glucose conditions at both 24 and 48 h (Figure 5A,B). This renoprotective effect was observed across a broad concentration range (31.25–1000 µg/mL) and restored cell viability to levels comparable to or slightly exceeding those observed under normal glucose conditions. Importantly, the protective effect did not increase linearly with concentration, suggesting a hormetic response in which low to moderate doses exert maximal cytoprotective activity. These findings indicate that beetroot spent extract effectively protects renal tubular cells against high-glucose-induced cytotoxicity.

## 4. Discussion

This study demonstrates that beetroot spent extracts possess strong antioxidant activity and exert a protective effect on renal tubular cells exposed to hyperglycemic stress. These findings highlight the biological value of beetroot processing by-products and support their potential application as natural dietary or functional ingredients for mitigating diabetes-related kidney damage [23]. Bioactive compounds such as phenolics, flavonoids, and betalains are often retained within cellular matrices or complex fiber structures [24,25]. As a result, these compounds can accumulate in the solid residue (spent material) generated during juice processing, leading to their enrichment relative to beetroot juice itself. Among the extracts evaluated, the hot water extract of beetroot spent material exhibited superior antioxidant activity when compared with the 70% ethanolic extract, as demonstrated by its higher ABTS radical scavenging capacity. This observation suggests that aqueous extraction is particularly effective in preserving and recovering hydrophilic antioxidant compounds, including betalains, which are known to be sensitive to organic solvents and high-ethanol concentrations [26,27]. The enhanced radical scavenging activity of the water extract is therefore consistent with the stability and extractability of these bioactive compounds in aqueous systems [28]. In contrast, high-ethanolic concentrations may reduce the recovery or stability of betalains and certain hydrophilic phenolics. In agreement with our findings, Righi Pessoa da Silva et al. reported that the extraction of beetroot using 100% ethanol significantly reduced the levels of betacyanin and betaxanthin [29], resulting in a decrease in free radical scavenging, as was measured in the ABTS assay [30].

Untargeted high-resolution mass spectrometry is widely used for comprehensive phytochemical screening; however, definitive quantification requires targeted HPLC-MS/MS analysis with authentic reference standards, which was beyond the scope of this study. In this work, UHPLC-ESI-QTOF-MS analysis revealed a diverse and complex phytochemical profile in the beetroot spent water extract, including putatively annotated flavonoids (e.g., quercetin, epicatechin, and gallocatechin), phenolic acids (e.g., caffeic acid, chlorogenic acid, and ferulic acid), and anthocyanin-related compounds. Many of these phytochemicals have been widely reported to exert antioxidant, anti-inflammatory, and nephroprotective effects through ROS scavenging, modulating redox-sensitive signaling pathways, and preserving mitochondrial function [31,32]. Definitive structural confirmation of individual phytochemicals would require targeted MS/MS experiments or nuclear magnetic resonance spectroscopy with authentic reference standards, which was beyond the scope of the present untargeted profiling study.

The stronger ABTS radical scavenging activity observed for beetroot juice and the hot water extract, compared with the 70% ethanolic extract, can be attributed to the preferential extraction of hydrophilic antioxidant activity of aqueous extracts rich in betalains and water-soluble phenolic compounds. Betalains are major contributors to beetroot antioxidant capacity and are highly polar molecules that exhibit greater stability and extractability in aqueous systems. Furthermore, the ABTS assay is more responsive to hydrophilic radical scavengers, which may further explain the lower apparent activity of the ethanolic extract despite the presence of phenolic compounds. Thus, these factors explain the lower apparent antioxidant activity of the ethanolic extract despite the presence of phenolic compounds.

The hot water extract of beetroot spent material improved cell viability in PBMC, SH-SY5Y, HEK-293, and MDA-MB-231 cells. However, the longer the culture period, the more it may exhibit a hormetic effect, with pro-oxidants emerging after prolonged exposure, especially in the presence of metal ions [33]. Furthermore, the extract may affect the pH or nutrient composition in the medium culture after being cultured for more than 48 h, resulting in decreased cell survival [34]. Prolonged exposure to high glucose levels in HEK-293 cells, which mimics hyperglycemia conditions, would reduce cell viability by increasing ROS production and mitochondrial dysfunction, thereby inducing oxidative stress [35]. These pathways can then lead to kidney cell damage, ultimately causing diabetic nephropathy [36].

Though a strictly dose-dependent relationship was not observed, beetroot spent extract consistently exerted a significant renoprotective effect across a defined concentration range. Such non-linear responses are characteristic of complex phytochemical mixtures and may reflect hormetic mechanisms, whereby low to moderate concentrations elicit cytoprotective effects, while higher concentrations provide no additional benefit or slightly reduce efficacy. Importantly, all effective concentrations significantly improved HEK-293 cell viability under high-glucose conditions, supporting the robustness of the renoprotective effect. Beetroot spent extract recovered the HEK-293 cells after prolonged exposure to high glucose, most likely due to its strong antioxidant capabilities. In addition to renal tubular cells, the cytotoxicity of beetroot spent extract was evaluated in SH-SY5Y neuronal cells and MDA-MB-231 breast cancer cells to assess general cellular tolerance and safety. The absence of cytotoxic effects across these distinct cell types supports the biocompatibility of the extract and suggests that its protective effects observed in HEK-293 cells are not due to nonspecific metabolic stimulation or toxicity.

The novelty of this study lies in its integrated approach combining untargeted high-resolution phytochemical profiling with in vitro renal cell-based evaluation of beetroot spent extract obtained from juice processing by-products. Unlike previous studies that primarily focused on beetroot juice or isolated compounds, this work demonstrates that beetroot spent material retains a diverse array of bioactive phytochemicals and exhibits cytoprotective effects in renal tubular cells under high-glucose-associated stress, highlighting its potential value as a functional ingredient derived from agricultural waste.

Although beetroot spent extract significantly improved renal tubular cell viability under hyperglycemic conditions and exhibited strong antioxidant activity in vitro, this study did not directly measure intracellular oxidative stress, apoptosis-related markers, or redox-regulated signaling pathways such as Nrf2/ARE. Consequently, the proposed antioxidant and cytoprotective mechanisms are speculative and inferred from the phytochemical profile and established bioactivities of phenolics, flavonoids, and betalains reported in the literature [9,37]. Collectively, these results indicate that beetroot spent extract effectively mitigates hyperglycemia-induced cytotoxicity in renal tubular cells and may help protect against oxidative stress-driven kidney injury. However, this study is limited by its in vitro design and the use of a single renal cell line. In addition, the study did not include an osmotic control (e.g., mannitol) to distinguish glucose-specific metabolic toxicity from hyperosmolar stress. Although high-glucose exposure is commonly used to model hyperglycemia-induced cellular injury, increased osmolarity may contribute to the observed cytotoxic effects. Therefore, the protective effects observed in this study should be interpreted as mitigation of high-glucose-associated stress, rather than exclusively glucose-specific toxicity. Future studies incorporating osmotic controls will be necessary to clarify this distinction.

Further studies incorporating direct assessments of ROS levels, mitochondrial function, apoptosis signaling, and antioxidant pathway activation are required to elucidate the molecular mechanisms underlying the observed renoprotective effects.

## 5. Conclusions

Beetroot spent water extract is a rich source of phenolic compounds, flavonoids, and betalains with strong antioxidant activity. The observed renoprotective effects may be associated with the antioxidant capacity of beetroot spent extract, as supported by its phytochemical composition and in vitro radical scavenging activity. These findings suggest that beetroot spent extract exerts cytoprotective effects in renal tubular cells under high-glucose-associated stress conditions in vitro. Further in vivo and clinical studies are required to validate these protective effects and facilitate the development of beetroot spent extract as a functional food ingredient or nutraceutical.

## Figures and Tables

**Table 1:** Total amounts of fresh beetroot, beetroot juice, spent beetroot, and spent extracts obtained through hot water and 70% ethanol extraction methods.

Beetroot Sample	Fresh Weight (kg)	Dried Weight (g)	Lyophilized Powder (g)	Yield (%)
Fresh beetroot	6.27	-	-	-
Juice	3.13	-	-	-
Spent	3.14	120.93	-	-
Water extract of spent		20.18	3.07	15.20
70% Ethanolic extract of spent		20.25	2.98	14.71

**Table 2:** TPC, TFC and TBC of beetroot juice and beetroot spent extracts prepared using hot water and 70% ethanol. Data are presented as mean ± SD values (*n* = 3).

Beetroot Sample	TPC		TFC		TBC	
	(mg GAE/g DW)	(mg GAE/g WW)	(mg QE/g DW)	(mg QE/g WW)	(mg/g DW)	(mg/g WW)
Juice	12.27 ± 3.03	0.24 ± 0.01	0.52 ± 0.11	0.01 ± 0.00	4.55 ± 0.01	90 ± 2
Beetroot spent						
Water extract	9.16 ± 1.08	1.56 ± 0.24	4.76 ± 0.37	0.86 ± 0.10	0.60 ± 0.00	91 ± 1
70% Ethanolic extract	5.41 ± 0.72	0.77 ± 0.07	0.29 ± 0.05	0.03 ± 0.02	0.28 ± 0.00	42 ± 1

## Data Availability

The original contributions presented in this study are included in the article/Supplementary Material. Further inquiries can be directed to the corresponding author.

## Supplementary Materials

The following supporting information can be downloaded at: https://www.mdpi.com/article/10.3390/foods15040769/s1, Table S1. Putative annotation of phytochemical constituents in hot water extract of beetroot spent material analyzed by UHPLC-ESI-QTOF-MS.

## Abbreviations/Symbols

The following abbreviations/symbols are used in this manuscript:

ABTS	2,2′-Azino-bis(3-ethylbenzothiazoline-6-sulfonic acid
ANOVA	Analysis of variance
DI	Deionized
DMSO	Dimethyl sulfoxide
DW	Dry weight
FBS	Fetal bovine serum
GA	Gallic acid
GAE	Gallic acid equivalent
l	Light path
MW	Molecular weight
MSI	Metabolic Standard Initiative
MTT	3-(4,5-Dimethylthiazol-2-yl)-2,5-diphenyltetrazolium bromide
OD	Optical density
PBMC	Peripheral blood mononuclear cell
Q	Quercetin
QE	Quercetin equivalent
ROS	Reactive oxygen species
SD	Standard deviations
TBC	Total betalain content
TFC	Total flavonoid content
TPC	Total phenolic content
Trolox	6-Hydroxy-2,5,7,8-tetramethylchroman-2-carboxylic acid
UHPLC-ESI-QTOF-MS	Ultra-high pressure liquid chromatography–electrospray ionization–quadrupole time-of-flight mass spectrometry
WW	Wet weight
ε	Molar extinction coefficient

## References

[1] Forbes J.M., Coughlan M.T., Cooper M.E. (2008). Oxidative stress as a major culprit in kidney disease in diabetes. Diabetes.

[2] Sun Y., Jin D., Zhang Z., Zhang Y., Zhang Y., Kang X., Jiang L., Tong X., Lian F. (2023). Effects of antioxidants on diabetic kidney diseases: Mechanistic interpretations and clinical assessment. Chin. Med..

[3] Sulkowski L., Matyja A., Matyja M. (2024). Social support and quality of life in hemodialysis patients: A comparative study with healthy controls. Medicina.

[4] Azeredo H. (2009). Betalains: Properties, sources, applications, and stability—A review. Int. J. Food Sci. Technol..

[5] Esatbeyoglu T., Ewald P., Yasui Y., Yokokawa H., Wagner A.E., Matsugo S., Winterhalter P., Rimbach G. (2016). Chemical characterization, free radical scavenging, and cellular antioxidant and anti-inflammatory properties of a stilbenoid-rich root extract of *Vitis vinifera*. Oxidative Med. Cell. Longev..

[6] Esatbeyoglu T., Wagner A.E., Schini-Kerth V.B., Rimbach G. (2015). Betanin—A food colorant with biological activity. Mol. Nutr. Food Res..

[7] Reddy M.K., Alexander-Lindo R.L., Nair M.G. (2005). Relative inhibition of lipid peroxidation, cyclooxygenase enzymes, and human tumor cell proliferation by natural food colors. J. Agric. Food Chem..

[8] Martinez R.M., Melo C.P.B., Pinto I.C., Mendes-Pierotti S., Vignoli J.A., Verri W.A., Casagrande R. (2024). Betalains: A narrative review on pharmacological mechanisms supporting the nutraceutical potential towards health benefits. Foods.

[9] Clifford T., Howatson G., West D.J., Stevenson E.J. (2015). The potential benefits of red beetroot supplementation in health and disease. Nutrients.

[10] Abdurrahman A., Adamu A.N., Ashimi A., Adekunle O.O., Bature S.B., Aliyu L.D., Akeem O., Abdullahi H., Lavin T., Daneji S. (2024). Predictors, prevalence and outcome of hypertensive disorders in pregnancy in Nigerian tertiary health facilities. BJOG.

[11] Akeem Olalekan A. (2024). The potential role of beetroot juice in hyperglycemia management: A review of mechanisms and clinical outcomes. Afr. J. Agric. Food Sci..

[12] Garnacho-Castano M.V., Pleguezuelos-Cobo E., Berbel M., Irurtia A., Carrasco-Marginet M., Castizo-Olier J., Veiga-Herreros P., Faundez-Zanuy M., Serra-Paya N. (2024). Effects of acute beetroot juice intake on performance, maximal oxygen uptake, and ventilatory efficiency in well-trained master rowers: A randomized, double-blinded crossover study. J. Int. Soc. Sports Nutr..

[13] Wang J., Jayaprakasha G.K., Patil B.S. (2020). UPLC-QTOF-MS fingerprinting combined with chemometrics to assess the solvent extraction efficiency, phytochemical variation, and antioxidant activities of *Beta vulgaris* L.. J. Food Drug Anal..

[14] Ainsworth E.A., Gillespie K.M. (2007). Estimation of total phenolic content and other oxidation substrates in plant tissues using Folin–Ciocalteu reagent. Nat. Protoc..

[15] Petry R.D., Ortega G.G., Silva W.B. (2001). Flavonoid content assay: Influence of the reagent concentration and reaction time on the spectrophotometric behavior of the aluminium chloride--flavonoid complex. Pharmazie.

[16] Shakir B.K., Vincenzi S. (2024). Estimation of betalain content in beetroot peel powder. Ital. J. Food Sci..

[17] Re R., Pellegrini N., Proteggente A., Pannala A., Yang M., Rice-Evans C. (1999). Antioxidant activity applying an improved ABTS radical cation decolorization assay. Free Radic. Biol. Med..

[18] Moghadam D., Zarei R., Tatar M., Khoshdel Z., Mashayekhi F.J., Naghibalhossaini F. (2022). Anti-proliferative and anti-telomerase effects of blackberry juice and berry- derived polyphenols on HepG2 liver cancer cells and normal human blood mononuclear cells. Anti-Cancer Agents Med. Chem..

[19] Denizot F., Lang R. (1986). Rapid colorimetric assay for cell growth and survival. Modifications to the tetrazolium dye procedure giving improved sensitivity and reliability. J. Immunol. Methods.

[20] Esmaeili S., Motamedrad M., Hemmati M., Mehrpour O., Khorashadizadeh M. (2019). Prevention of kidney cell damage in hyperglycaemia condition by adiponectin. Cell Biochem. Funct..

[21] Gest C., Joimel U., Huang L., Pritchard L.L., Petit A., Dulong C., Buquet C., Hu C.Q., Mirshahi P., Laurent M. (2013). Rac3 induces a molecular pathway triggering breast cancer cell aggressiveness: Differences in MDA-MB-231 and MCF-7 breast cancer cell lines. BMC Cancer.

[22] Lin M., Li L., Zhang Y., Zheng L., Xu M., Rong R., Zhu T. (2014). Baicalin ameliorates H_2_O_2_ induced cytotoxicity in HK-2 cells through the inhibition of ER stress and the activation of Nrf2 signaling. Int. J. Mol. Sci..

[23] Kujala T.S., Loponen J.M., Klika K.D., Pihlaja K. (2000). Phenolics and betacyanins in red beetroot (*Beta vulgaris*) root:  Distribution and effect of coldstorage on the content of total phenolics and three individual compounds. J. Agric. Food Chem..

[24] Kujala T., Loponen J., Pihlaja K. (2014). Betalains and phenolics in red beetroot (*Beta vulgaris*) peel extracts: Extraction and characterisation. Z. Naturforsch. C J. Biosci..

[25] Afzaal M., Saeed F., Ahmed A., Khalid M.A., Islam F., Ikram A., Hussain M., Fareed F., Anjum W. (2022). Red beet pomace as a source of nutraceuticals. Food and Agricultural Byproducts as Important Source of Valuable Nutraceuticals.

[26] Strack D., Vogt T., Schliemann W. (2003). Recent advances in betalain research. Phytochemistry.

[27] Stoica F., Râpeanu G., Rațu R.N., Stănciuc N., Croitoru C., Țopa D., Jităreanu G. (2025). Red beetroot and its by-products: A comprehensive review of phytochemicals, extraction methods, health benefits, and applications. Agriculture.

[28] Kujala T.S., Vienola M.S., Klika K.D., Loponen J.M., Pihlaja K. (2002). Betalain and phenolic compositions of four beetroot (*Beta vulgaris*) cultivars. Eur. Food Res. Technol..

[29] Righi Pessoa da Silva H., da Silva C., Bolanho B.C. (2018). Ultrasonic-assisted extraction of betalains from red beet (*Beta vulgaris* L.). J. Food Process Eng..

[30] Eyshi S., Ghareaghajlou N., Afshar Mogaddam M.R., Ghasempour Z. (2024). Red beet betalains extraction process: A comprehensive review of methods, applications, and physicochemical properties. Food Sci. Nutr..

[31] El Gamal A.A., AlSaid M.S., Raish M., Al-Sohaibani M., Al-Massarani S.M., Ahmad A., Hefnawy M., Al-Yahya M., Basoudan O.A., Rafatullah S. (2014). Beetroot (*Beta vulgaris* L.) extract ameliorates gentamicin-induced nephrotoxicity associated oxidative stress, inflammation, and apoptosis in rodent model. Mediat. Inflamm..

[32] Baião D.D.S., Silva D., Paschoalin V.M.F. (2020). Beetroot, a remarkable vegetable: Its nitrate and phytochemical contents can be adjusted in novel formulations to benefit health and support cardiovascular disease therapies. Antioxidants.

[33] Calabrese E.J., Mattson M.P. (2011). Hormesis provides a generalized quantitative estimate of biological plasticity. J. Cell Commun. Signal..

[34] Freshney R.I. (2015). Culture of Animal Cells: A Manual of Basic Technique and Specialized Applications.

[35] Kang B.P., Frencher S., Reddy V., Kessler A., Malhotra A., Meggs L.G. (2003). High glucose promotes mesangial cell apoptosis by oxidant-dependent mechanism. Am. J. Physiol. Renal Physiol..

[36] Forbes J.M., Cooper M.E. (2013). Mechanisms of diabetic complications. Physiol. Rev..

[37] Khan M.I. (2016). Stabilization of betalains: A review. Food Chem..

