# Supplementary material for: Renoprotective Potential of Beetroot Spent Extract Under Hyperglycemic Conditions
Source: Foods. 2026 Feb 20;15(4):769. doi: 10.3390/foods15040769 (PMC12940966; doi:10.3390/foods15040769)
Supplement: Supplementary file 1 [file foods-15-00769-s001.zip › foods-4114949-supplementary.pdf]

Supplementary Table

Table S1. Putative annotation of phytochemical constituents in hot water extract of beetroot spent material analyzed by using UHPLC-ESI-QTOF-MS.

Time (min)	Mass (g/mol) Reference	Observed	Error (ppm)	Molecular formula	Putative compounds/ compound class
1.429	128.047	128.046	-9.45	$C_6H_8O_3$	Dihydrophloroglucinol
1.485	208.074	208.075	8.1	$C_{11}H_{12}O_4$	5-(3',4'-dihydroxyphenyl)-γ-valerolactone
1.617	358.090	358.090	0.56	$C_{15}H_{18}O_{10}$	Dihydrocaffeic acid 3-O-glucuronide
1.628	458.085	458.086	2.35	$C_{22}H_{18}O_{11}$	Epigallocatechin gallate
1.684	126.032	126.031	-3.47	$C_6H_6O_3$	Phloroglucinol
1.689	247.999	247.998	-3.52	$C_8H_8O_7S$	Vanillic acid derivative
1.783	302.043	302.044	3.06	$C_{15}H_{10}O_7$	Quercetin
1.783	259.999	259.999	1.1	$C_9H_8O_7S$	Caffeic acid 3-sulfate
1.839	164.047	164.049	9.32	$C_9H_8O_3$	p-Coumaric acid
2.038	138.032	138.031	-8.19	$C_7H_6O_3$	2,5-Dihydroxybenzaldehyde
2.060	306.074	306.075	2.36	$C_{15}H_{14}O_7$	(+)-Gallocatechin
2.082	170.022	170.021	-2.48	$C_7H_6O_5$	Gallic acid
2.121	326.100	326.099	-1.58	$C_{15}H_{18}O_8$	trans-p-Coumaric acid 4-glucoside
2.121	474.043	474.044	0.53	$C_{21}H_{14}O_{13}$	m-Trigallic acid
2.126	164.047	164.047	-1.45	$C_9H_8O_3$	p-Coumaric acid
2.126	356.074	356.074	0.22	$C_{15}H_{16}O_{10}$	Caffeic acid 3-O-glucuronide
2.132	259.999	259.999	3.05	$C_9H_8O_7S$	Caffeic acid conjugate
2.137	180.042	180.041	-4.93	$C_9H_8O_4$	Caffeic Acid
2.148	306.038	306.038	1.84	$C_{14}H_{10}O_8$	2- Protocatechoyl phloroglucinolcarboxylate
2.298	290.079	290.078	-3.7	$C_{15}H_{14}O_6$	Epicatechin isomer
2.375	478.148	478.151	6.96	$C_{23}H_{26}O_{11}$	4'-O-Methyl-(-)- epicatechin-5-O-β-glucuronide
2.795	354.095	354.094	-2.05	$C_{16}H_{18}O_9$	Chlorogenic acid isomer I
3.691	354.095	354.096	3.22	$C_{16}H_{18}O_9$	Chlorogenic acid isomer II
4.062	290.079	290.081	8.25	$C_{15}H_{14}O_6$	Epicatechin-related isomer
4.167	452.132	452.130	-3.53	$C_{21}H_{24}O_{11}$	Catechin 5-glucoside
4.581	493.135	493.134	-1.2	$C_{23}H_{25}O_{12}$	Malvidin 3-galactoside
4.792	240.099	240.099	-4.38	$C_{12}H_{16}O_5$	Isopropyl 3-(3,4- dihydroxyphenyl)-2-hydroxypropanoate
4.935	322.069	322.069	0.09	$C_{15}H_{14}O_8$	Gallocatechin-4β-ol
5.964	226.084	226.082	-8.39	$C_{11}H_{14}O_5$	Dihydrosinapic acid
6.058	286.084	286.084	-0.89	$C_{16}H_{14}O_5$	Dihydrobiochanin A
6.157	507.114	507.115	1.86	$C_{23}H_{23}O_{13}$	Delphinidin glycoside
6.50	224.068	224.067	-6.94	$C_{11}H_{12}O_5$	cis-Sinapic acid
6.694	152.047	152.046	-9.66	$C_8H_8O_3$	2-Hydroxyphenylacetic acid
6.843	386.100	386.099	-2.28	$C_{20}H_{18}O_8$	8-8'-Dehydrodiferulic acid
6.865	626.148	626.148	-0.10	$C_{27}H_{30}O_{17}$	Quercetin glycoside
6.926	610.153	610.154	0.53	$C_{27}H_{30}O_{16}$	Quercetin di-hexoside
7.064	330.038	330.036	-3.93	$C_{16}H_{10}O_8$	2,3-Di-O-methylellagic acid
7.098	290.079	290.078	-4.09	$C_{15}H_{14}O_6$	Flavane-3-ol (epicatechin-type)

7.440	610.153	610.154	0.69	$C_{27}H_{30}O_{16}$	Quercetin di-hexoside
7.446	611.161	611.158	-4.47	$C_{27}H_{31}O_{16}$	Anthocyanidin glycoside
8.342	194.058	194.059	7.69	$C_{10}H_{10}O_4$	Ferulic acid
9.403	260.105	260.104	-2.97	$C_{15}H_{16}O_4$	3-(1,1-Dimethylallyl)scopoletin
9.785	541.913	541.914	1.68	$C_{15}H_{10}O_{16}S_3$	Quercetin 3,7,4'-tri-O-sulfate
10.062	304.095	304.097	8.86	$C_{16}H_{16}O_6$	4'-O-Methylcatechin
10.084	422.158	422.158	1.60	$C_{21}H_{26}O_9$	3-(1,1-Dimethylallyl)scopoletin 7-glucoside
10.554	358.090	358.089	-1.37	$C_{15}H_{18}O_{10}$	Dihydrocaffeic acid 3-O-glucuronide
10.758	190.099	190.098	-8.46	$C_{12}H_{14}O_2$	3-Dimethylallyl-4-hydroxybenzaldehyde
11.107	390.204	390.201	-7.48	$C_{22}H_{30}O_6$	6,7-Dimethoxy-7-epirosmanol
13.838	194.058	194.058	2.26	$C_{10}H_{10}O_4$	Ferulic acid
16.156	318.110	318.109	-4.73	$C_{17}H_{18}O_6$	4',7-Di-O-methylcatechin
17.665	486.116	486.119	5.37	$C_{24}H_{22}O_{11}$	4'-Methyl-(-)-epigallocatechin 3-(4-methyl-gallate)
17.676	789.188	789.189	1.26	$C_{36}H_{37}O_{20}$	Delphinidin 3-glucoside 5-caffoyl-glucoside
20.27	316.116	316.116	0.99	$C_{14}H_{20}O_8$	Hydroxytyrosol 1-O-glucoside
21.790	262.015	262.016	2.78	$C_9H_{10}O_7S$	Dihydrocaffeic acid 3-sulfate
23.378	479.119	479.118	-3.02	$C_{22}H_{23}O_{12}$	4'-O-Methyldelphinidin 3-O-β-D-glucoside
35.638	817.240	817.239	-1.15	$C_{35}H_{45}O_{22}$	Malvidin 3-sophoroside 5-glucoside
37.678	646.174	646.174	-0.58	$C_{27}H_{34}O_{18}$	Leucodelphinidin 3-[galactosyl-(1→4)-glucoside]
38.037	594.137	594.133	-7.45	$C_{30}H_{26}O_{13}$	Epicatechin-(4β→8)-gallocatechin isomer I
38.989	594.137	594.136	-1.56	$C_{30}H_{26}O_{13}$	Epicatechin-(4β→8)-gallocatechin isomer II

Note: Compounds detected with identical accurate mass and molecular formula but different retention times are reported as separate putative isomers (Metabolic Standard Initiative Level 2). These features may represent positional, stereochemical, or conformational isomers. Definitive structural assignment requires MS/MS fragmentation analysis and/or comparison with authentic reference standards.
